# Collective Call to Action for HIV/AIDS Community-Based Collaborative Science in the Era of COVID-19

**DOI:** 10.1007/s10461-020-02860-y

**Published:** 2020-04-16

**Authors:** Steven Shoptaw, David Goodman-Meza, Raphael J. Landovitz

**Affiliations:** 1 Department of Family Medicine, David Geffen School of Medicine at UCLA, Los Angeles, CA USA; 2 Division of Infectious Diseases, David Geffen School of Medicine at UCLA, Los Angeles, CA USA

We are fighting the threat of a new, frightening pandemic that is changing lives in ways that were unimaginable. As individuals try to make sense of the COVID-19 crisis and take care of their loved ones, it is easy for the public to forget about co-occurring epidemics of opioids, sexually transmitted infections (STIs), viral hepatitis, gun violence, and incarceration to name a few. COVID-19 is eroding already-strained health systems—in addition to further reducing its capacity to adequately address HIV/AIDS prevention and care—and associated endemics of STIs.

To respond, COVID-19 mandates the tactic of re-purposing resources. The expertise, including the intellectual and material resources brought to the HIV/AIDS fight by nurses, emergency medicine, hospitalists, infectious disease and intensive care clinicians are now whipsawed to the front lines for the overwhelming treatment response to COVID-19 response. Providers from primary care and allied health are also being called. Physicians have reminded us that one cost to this tactic is that some health professionals will not be with us on the other side of this crisis. That realization is jarring—but it is a reminder of the thread of humanity that intertwines with this pandemic, illustrated by our professional and personal connections. Trickle-down caring should be as highly prioritized as trickle-down economics.

If there is any lesson we are learning, it is that the exponential increases in sick and dying citizens underscores the point that epidemic dynamics are highly predictable [1, 2]. It is also predictable that the costs of tactical decisions needed now to respond to COVID-19 can be calculated, modeled, and paid for over decades to come [3]. Modelers may use different inputs, but what is unanimous is that re-purposing of scientific, clinical and material resources takes critical partner hands off the levers that were maintaining control of HIV for those living with the disease and to prevent transmission of HIV to others.

As recently as late January, we at UCLA^1^ hosted a meeting of public health and community leaders from all eight impacted California counties to plan transformation of our collective HIV prevention and treatment efforts and to re-commit to the efforts needed to End the HIV Epidemic. The goals of the meeting were: to identify barriers and facilitators to sharing and scaling-up HIV prevention across the counties; to examine techniques for regional HIV surveillance to identify “hot spots”/micro-epidemics to guide allocation of prevention resources and trim the outbreak; to engage stakeholders and policy makers to prepare for long-acting injectable medications. Then COVID-19 shifted all of our work; we need now to integrate into our objectives shifts that accommodate responses needed for COVID-19 and to maintain commitments to make measurable progress not only to maintain gains in HIV treatment and prevention, but to end the HIV epidemic.

## Undetectable is Untransmittable

First, we know that HIV is not transmitted when people living with HIV keep their viral levels undetectable [4]. Yet remaining undetectable depends on regular access to blood tests to show medications are working. As machines and people who conduct these tests are re-purposed for COVID-19, fewer tests for HIV viral load will be available. Fewer clinicians will have time to meet with patients. Patients will be less certain of their HIV status. This threatens hard fought scientific advancements, including treatment as prevention [5]. Now is the time to triage viral load tests, and prioritize tests for patients with histories of poorly controlled levels. Tele-health visits for patients with histories of consistently undetectable levels can preserve valuable clinician resources. We know how to do this, but never before at scale. Let us try to do that now, and study the process as we do. Work is already underway by University of Pennsylvania researchers^2^ to work with the public health department and community to evaluate the most effective existing and new U=U messaging. A similar effort has been initiated by Yale investigators in Puerto Rico.^3^ This work continues and could be undertaken in many more communities.

## HIV Testing and Counseling

HIV prevention is built upon free and accessible HIV antibody testing and counseling—particularly in neighborhoods and communities disproportionally affected by HIV. Today, most of these venues are closed for safety reasons. Unfortunately, the need for these testing services is unchanged. Moreover, in-person testing in clinics and emergency rooms is all but impossible as infectious disease expertise is shifted toward COVID-19. The costs for even temporary stoppage of freely available HIV testing risks new, undetected HIV infections—backsteps our communities cannot afford. Science shows that most new HIV transmissions are linked to people who become newly infected but are unaware of their HIV positive status [6, 7]—just like with COVID-19. Until venue-based testing is back in full effect, now is the time to scale-up access to free or low-cost in-home testing kits. On-line web pages on sexual health can be developed easily in collaboration with individuals who have lost their jobs or companies who want to be altruistic, to distribute kits. Yale-funded researchers are partnering with collaborators in Puerto Rico to learn how best to ramp up home HIV-testing, never more important. In Miami,^4^ early-stage investigators have launched a project to provide free HIV testing and same-day start packet for HIV medications, in mobile syringe services programs. A UCSF project team^5^ is already working on implementation of a sexual health model for rural Sacramento County, California. They aim to adapt an evidence-based sexual health services intervention designed to increase PrEP uptake to fit the local HIV epidemic in Sacramento County where racial and ethnic minority populations are disproportionately affected by HIV. It’s important to duplicate this work in high-need jurisdictions around the country.

## HIV Pre-exposure Prophylaxis (PrEP)

PrEP is effective and requires consultation with a medical clinician and clearance from labs [8]. Though PrEP is available at any medical office, most patients get PrEP from infectious disease and primary care clinicians comfortable and effective in prescribing PrEP [9]. These challenges are magnified as clinicians who prescribe PrEP are called to manage COVID-19. Tele-health companies already have solved this problem and can provide PrEP remotely [10]. Pharmacies are being considered for dispensing PrEP already, with potential for providing this service with minimal clinical resource [11, 12]. These critical resources need to be scaled quickly. We need to identify who will lead this effort, and use our discretionary time to organize a massive push on this front. There are already models to draw from. University of Miami researchers and Latinos Salud, a South Florida-based Latino MSM HIV-agency are working with Walgreens, CVS, Navarro and Target pharmacies to build an HIV Pharmacy Network to reach Latino men in Miami Dade county. And more: Now is the time to build academic and community partnerships in preparing for implementation of low-threshold use of long-acting injectable (LAI) ART as HIV prevention in anticipation of likely FDA approval and commercial availability [13].

## Treatment of Sexually Transmitted Infections (STIs)

STIs, especially syphilis, co-occur with HIV [14]. Lab machines that test for STDs are different than those used to test for COVID-19, but the clinics and clinicians who test and treat STDs are involved heavily in the fight against COVID-19. This risks further the release of a key lever to diagnose and treat STIs and HIV. Now is the time to broaden venues for STI testing to involve primary care, addiction treatment settings, or anywhere that makes sense in each jurisdiction. This may require considerate and strategic communication with policymakers and community leaders to keep attention to STIs in this era of COVID-19. Strategy sessions now can help re-commit to keeping free or low-cost STI testing as the bellwether of sexual health in our communities.

## Comorbidities and Disparities

Persons living with HIV or who live in communities disproportionally affected by HIV often also are grappling with combinations of mental health issues, substance use disorder(s) and barriers to social determinants of health, including unstable housing and incarceration, and multiple intersecting stigmas and discrimination [15]. These individuals have long-standing challenges to managing HIV [16] and to persisting in HIV prevention [17], and sadly some of these may even be magnified during this pandemic. Differential costs for redirecting resources to COVID-19 include leaving further behind those who live with these comorbidities, including increasing numbers of those living with consistently high viral counts, of those who cannot sustain PrEP use, and consequently with the number of those who become HIV-infected. Now is the time to scale-up innovative, technology-driven, community-based prevention outreach and HIV care for those who are living with these comorbidities of mental health, substance use disorders, and barriers to social determinants of health.

Researchers at Columbia University^6^ in New York City, a place reeling from COVID-19, are already building a coalition to overcome intersecting stigmas and improve HIV prevention, care access, and health outcomes. The objective of this activity is to identify where and how stigma-reduction interventions might most optimally be implemented and to explore how the promotion of resilience might contribute to this process. To accomplish this objective, the HIV Center, the NYC Department of Health and Mental Hygiene, the New York State DOH, and the Northeast/Caribbean AIDS Education and Training Center have established a partnership to establish the NYC Stigma and Resilience Coalition, a multi-sector, interdisciplinary coalition of HIV-related organizations, affected communities, non-traditional partners, public health officials and academic researchers, to devise strategies for overcoming HIV and related stigmas.

## Harm Reduction

Finally, provision of harm reduction supplies (e.g., sterile drug use equipment) are essential to preventing HIV, STIs and hepatitis C transmission [18]. With shelter-in-place orders, again, access to these supplies is severely limited. On the other hand, shelter-in-place orders leave lots of time to fill. People will have sex and use drugs to pass the time and in ways that are also entirely predictable and understandable, but that also confer risks for HIV infection. But do we know this? Let’s ask them. There are many NIH-funded ongoing studies with cohorts of HIV negative and HIV positive participants who also stand at the ready to help, and with time on their hands! Let’s ask them important questions and use their responses to help those in their community who are not as fortunate to be in such studies. Without access to harm reduction supplies, new HIV infections (and STIs and Hepatitis-C)—are unavoidable. Now is the time for pharmacies to ensure access to syringes, for all of us to place condoms and lube anywhere people are still congregating. Another round of NIH supplements funded through the End the HIV Epidemic: A Plan for America Initiative [19] includes inspiring work to reach African-Americas in five venues in five Miami Dade zip codes with the highest number of Black individuals living with HIV: (1) barbershops, (2) hair/beauty salons, (3) laundromats, (4) corner stores, and (5) mechanics. In many other communities, we do not know whether, where, and how people are still meeting up to share their fears and get social support, and some semblance of normalcy. We should find out, and reach them there.

COVID-19 is changing the lives of the people in clinical care, our research participants, and our research teams. We must act now, and act smart to maintain the attention, expertise, resources, novel collaborations, advocacy, community engagement, research, and press on the predictable surge in new infections of HIV, STIs and Hepatitis C—linked to needed efforts to contain COVID-19.

## Footnotes

1 P30 MH058107; Center for HIV Identification Prevention and Treatment Services.

2 P30 MH097488; Penn Mental Health AIDS Research Center.

3 P30 MH062294; Center for Interdisciplinary Research on AIDS.

4 P30 MH118043; University of Miami Developmental HIV/AIDS Mental Health Research Center.

5 P30 MH062246; Center for AIDS Prevention Studies.

6 P30 MH043520; HIV Center for Clinical and Behavioral Studies.

## References

[1] Li Q, Guan X, Wu P, Wang X, Zhou L, Tong Y (2020). Early transmission dynamics in Wuhan, China, of novel coronavirus-infected pneumonia. N Engl J Med..

[2] Zhao S, Chen H. Modeling the epidemic dynamics and control of COVID-19 outbreak in China. Quantitative Biol. 2020;1–9. 10.1007/s40484-020-0199-0 PMC7095099 32219006

[3] Verity R, Okell LC, Dorigatti I, Winskill P, Whittaker C, Ferguson NM. Estimates of the severity of coronavirus disease 2019: a model-based analysis. Lancet Infect Dis. 2020. 10.1016/S1473-3099(20)30243-7 PMC7158570 32240634

[4] Eisinger RW, Dieffenbach CW, Fauci AS (2019). HIV viral load and transmissibility of HIV infection: undetectable equals untransmittable. JAMA-J Am Med Assoc..

[5] Cohen MS, Chen YQ, McCauley M, Gamble T, Hosseinipour MC, Kumarasamy N, HPTN 052 Study Team (2011). Prevention of HIV-1 infection with early antiretroviral therapy. N Engl J Med..

[6] Hall HI, Holtgrave DR, Maulsby C (2012). HIV transmission rates from persons living with HIV who are aware and unaware of their infection. Aids..

[7] Skarbinski J, Rosenberg E, Paz-Bailey G, Hall HI, Rose CE, Viall AH (2015). Human immunodeficiency virus transmission at each step of the care continuum in the United States. JAMA Intern Med..

[8] Fonner VA, Dalglish SL, Kennedy CE, Baggaley R, O'Reilly KR, Koechlin FM (2016). Effectiveness and safety of oral HIV preexposure prophylaxis for all populations. Aids..

[9] Petroll AE, Walsh JL, Owczarzak JL, McAuliffe TL, Bogart LM, Kelly JA (2017). PrEP Awareness, familiarity, comfort, and prescribing experience among US primary care providers and HIV specialists. AIDS Behav..

[10] Touger R, Wood BR (2019). A Review of telehealth innovations for HIV pre-exposure prophylaxis (PrEP). Curr HIV/AIDS Rep..

[11] Tung EL, Thomas A, Eichner A, Shalit P (2018). Implementation of a community pharmacy-based pre-exposure prophylaxis service: a novel model for pre-exposure prophylaxis care. Sex Health..

[12] Crawford ND, Albarran T, Chamberlain A, Hopkins R, Josma D, Morris J, et al. Willingness to discuss and screen for pre-exposure prophylaxis in pharmacies among men who have sex with men. J Pharm Pract. 2020;897190020904590. 10.1177/0897190020904590 PMC10443399 32067554

[13] Clement ME, Kofron R, Landovitz RJ (2020). Long-acting injectable cabotegravir for the prevention of HIV infection. Curr Opin HIV AIDS..

[14] Berry SA, Ghanem KG, Mathews WC, Korthuis PT, Yehia BR, Agwu AL (1999). Brief report: gonorrhea and chlamydia testing increasing but still lagging in HIV clinics in the United States. J Acquir Immune Defic Syndr.

[15] Pantalone DW, Nelson KM, Batchelder AW, Chiu C, Gunn HA, Horvath KJ. A systematic review and meta-analysis of combination behavioral interventions co-targeting psychosocial syndemics and HIV-related health behaviors for sexual minority men. J Sex Res. 2020;1–28. 10.1080/00224499.2020.1728514 PMC7457381 32077326

[16] Padilla M, Frazier EL, Carree T, Luke Shouse R, Fagan J (2020). Mental health, substance use and HIV risk behaviors among HIV-positive adults who experienced homelessness in the United States—Medical Monitoring Project, 2009–2015. AIDS Care..

[17] Krakower D, Maloney KM, Powell VE, Levine K, Grasso C, Melbourne K (2019). Patterns and clinical consequences of discontinuing HIV preexposure prophylaxis during primary care. J Int AIDS Soc..

[18] Larney S, Peacock A, Leung J, Colledge S, Hickman M, Vickerman P (2017). Global, regional, and country-level coverage of interventions to prevent and manage HIV and hepatitis C among people who inject drugs: a systematic review. Lancet Glob Health..

[19] Azar A. HHS.gov2019. https://www.hhs.gov/blog/2019/02/05/ending-the-hiv-epidemic-a-plan-for-america.html.

